# Supplementary material for: MPLNet: Mamba prompt learning network for semantic segmentation of remote sensing images of traditional villages
Source: PLoS One. 2026 Feb 2;21(2):e0341130. doi: 10.1371/journal.pone.0341130 (PMC12863532; doi:10.1371/journal.pone.0341130)
Supplement: Attachment — Submitted filename: Reply to the comments10.13.pdf [file pone.0341130.s001.pdf]

Response to Reviewers – MPLNet

Zhang Cheng

October 2025

Reviewer #1 – Comment 1 (Language and grammar)

Comment (as received). "There are irregular expressions and grammatical errors in the manuscript."

Response. We carried out a careful, line-by-line edit. The goal was clarity and consistency rather than changing scientific content. In the following, we summarize what was standardized and where it was applied.

(1) Style and readability. Long or awkward sentences were rewritten into clear main clauses; informal wording was removed; articles and prepositions were corrected. Punctuation and parallel structures were normalized throughout the paper.

(2) Scope of claims. Anthropomorphic or overstated phrases were eliminated. Model behaviour is now described using reported metrics and concrete error patterns, with each claim tied to a specific figure or table.

(3) Terminology and abbreviations. All abbreviations are defined at first mention and then used consistently (e.g., TV-RSI-413, MPLNet, MFM, SS2D, SiLU). Capitalization and hyphenation follow a single style (e.g., "state-of-the-art", "tile-level").

(4) Tense and voice. Methods are written in the present tense; experiments and results in the past tense. Contribution bullets were rewritten to use parallel grammar and unambiguous subjects.

(5) Mathematics and units. Notation was unified (scalars italic, operators roman). SI spacing is used for units and sizes (e.g., 256×256). We state the NCHW layout and *float32* once and keep this consistent.

(6) Figures, tables, captions. Captions use sentence case, define abbreviations at first appearance, and avoid interpretation beyond evidence. Table headers use consistent metric names (mIoU, mAcc, Boundary-F1, Connectivity).

Representative corrections (before → after).

- "The location and the landscape pattern ... show a deep understanding of the environment." → "The location and the landscape pattern reflect cumulative adaptations to terrain, hydrology and land-use constraints."
- "We adopt many tricks, which is helpful." → "We use standard strong augmentations (random scale/flip/crop), which improved boundary recall in our experiments."

- "MFM can fully understand global and local features and therefore perceive any structure." → "MFM aggregates local and mid-range context; the gain is most visible on thin structures and class boundaries."
- "The public datasets Potsdam and Wassingen are used." → "We evaluate on ISPRS Potsdam and Vaihingen; dataset names and splits follow the official conventions."

Manuscript locations updated. Abstract; Introduction (final paragraph and contribution bullets); Dataset; Method; Experiments/Results; figure captions; and tables. We supply a clean version and a tracked-changes version. No scientific content was altered for this item.

Reviewer #1 – Comment 2 (Notation and dimensions)

Comment (as received). "The definitions of symbols in formulas are incomplete. For instance, 'C3S' in Eq. (1) is not explained; although r_i, P_i, and Q_i in Eqs. (5)–(6) are defined, key attributes such as their dimensions and data types are not specified."

Response. Thank you for pointing this out. We revised Sec. 3 to define operators and symbols in their first use and to explicitly state tensor shapes, data types, and broadcast rules. These edits are limited to notation and do not change the method or its results.

What we clarified: We insert a short paragraph after Eq. (1) that serves as a compact glossary. First, we define C3S(k, s, p) as a 2D convolution with kernel $k\times k$, stride s, and padding p, followed by BatchNorm and SiLU; unless otherwise stated, we use C3S$(3, 1, 1)$. Tensors adopt NCHW and *float32*. For ResNet-50 stages $i \in \{1, 2, 3, 4\}$ we set $C_i \in \{256, 512, 1024, 2048\}$ and

$$(H_i, W_i) \in \{(H/4, W/4), (H/8, W/8), (H/16, W/16), (H/32, W/32)\},$$

so for 256×256 tiles they become $(64, 64)$, $(32, 32)$, $(16, 16)$, $(8, 8)$. The frozen teacher branch produces $r_i \in \mathbb{R}^{B\times C_i\times H_i\times W_i}$; the trained student produces F_i of the same shape. When spatial sizes differ, r_i is aligned to F_i by bilinear resize and/or a 1×1 projection. We form a channel gate $P_i \in \mathbb{R}^{B\times C_i\times 1\times 1}$ (GAP + linear) and a spatial gate $Q_i \in \mathbb{R}^{B\times 1\times H_i\times W_i}$ (1×1 conv). Broadcasting is explicit: $\sigma(\cdot)$ is Sigmoid for P_i and $\phi(\cdot)$ is SiLU inside the injected branch. The gated residual update is

$$\tilde{F}_i \;=\; F_i \;+\; \sigma(P_i) \,\odot\, \phi\big(Q_i \odot \mathcal{M}(r_i)\big),$$

where $\odot$ is the multiplication of elements in a single element and $\mathcal{M}(\cdot)$ denotes the Mamba/SS2D block. Teacher parameters (backbone+decoder) are frozen; gradients flow through the student, $\mathcal{M}(\cdot)$, the gates, and the final decoder. Figure labels now mark "frozen" vs. "trainable" for clarity.

Exact manuscript insertion (after Eq. (1), Sec. 3).

> **Operator Glossary & Symbols.** C3S(k, s, p): 2D conv ($k\times k$, stride s, padding p) + BN + SiLU; default C3S$(3, 1, 1)$. Tensors use NCHW and float32. Stage i: $C_i \in \{256, 512, 1024, 2048\}$; (H_i, W_i) are $\{(H/4, W/4), (H/8, W/8), (H/16, W/16), (H/32, W/32)\}$. Teacher features $r_i \in \mathbb{R}^{B\times C_i\times H_i\times W_i}$ are aligned to student features F_i via bilinear resize and/or a 1×1 projection. Gates: $P_i \in \mathbb{R}^{B\times C_i\times 1\times 1}$ (channel) and $Q_i \in \mathbb{R}^{B\times 1\times H_i\times W_i}$ (spatial). Broadcasting gives
>
> $$\tilde{F}_i = F_i + \sigma(P_i) \,\odot\, \phi\big(Q_i \odot \mathcal{M}(r_i)\big),$$
>
> with σ = Sigmoid, ϕ = SiLU, and $\mathcal{M}(\cdot)$ the Mamba/SS2D block.

Reviewer #1 – Comment 3 (How Mamba yields multi-directional context)

Comment (as received). "Only 'intra-modal contextual modeling is performed via the Mamba mechanism' is mentioned. The manuscript does not explain how the Mamba mechanism achieves multi-directional contextual information."

Response. Thank you for raising this point. We clarified in Sec. 3.1 how the Mamba block inside MFM is used to aggregate context along *four* scan directions on the 2D feature map and how the directional outputs are fused. MFM remains strictly intra-modal (it only consumes the student features F_i); cross-modal cues are handled in the gated prompt branch as described in Comment 2.

Mechanism (concise). Given $F_i \in \mathbb{R}^{B\times C_i\times H_i\times W_i}$, we unfold it into four sequences by rasterizing along rows and columns in both orders: left→right (→), right→left (←), top→bottom (↓), bottom→top (↑). Each sequence is processed by a Mamba/SS2D block with shared hyperparameters (weights may be shared or per-direction; in our default we share weights to keep parameters minimal) and then reshaped back to (H_i, W_i):

$$\mathcal{M}_{\rightarrow}(F_i),\ \mathcal{M}_{\leftarrow}(F_i),\ \mathcal{M}_{\downarrow}(F_i),\ \mathcal{M}_{\uparrow}(F_i)\ \in\ \mathbb{R}^{B\times C_i\times H_i\times W_i}.$$

We concatenate and fuse the four directional responses and smooth them depthwise to reduce orientation-specific noise:

$$G_i\ =\ \mathrm{DWConv}_{3\times 3}\big(\,W_f * \mathrm{Concat}[\mathcal{M}_{\rightarrow}, \mathcal{M}_{\leftarrow}, \mathcal{M}_{\downarrow}, \mathcal{M}_{\uparrow}]\,\big),\quad G_i \in \mathbb{R}^{B\times C_i\times H_i\times W_i},$$

where W_f is a 1×1 projection back to C_i channels. The final update is a gated residual that prevents over-smoothing in flat regions and preserves structure along edges/lines:

$$\tilde{F}_i\ =\ F_i\ +\ \sigma(P_i)\ \odot\ \phi\big(Q_i \odot G_i\big),$$

with $P_i \in \mathbb{R}^{B\times C_i\times 1\times 1}$ (channel gate), $Q_i \in \mathbb{R}^{B\times 1\times H_i\times W_i}$ (spatial gate), σ the Sigmoid and ϕ the SiLU. Teacher parameters are frozen; gradients flow only through the student path, the Mamba blocks, the gates, and the decoder.

Exact manuscript insertion (Sec. 3.1, after introducing MFM).

> **Directional scans and fusion.** To capture context from multiple orientations on F_i, MFM applies a Mamba/SS2D block to four rasterizations of the feature map (left→right, right→left, top→bottom, bottom→top), reshapes the results back to (H_i, W_i), concatenates them, and projects with a 1×1 layer followed by depthwise 3×3 smoothing. The fused map G_i is injected through a gated residual $\tilde{F}_i = F_i + \sigma(P_i) \odot \phi(Q_i \odot G_i)$, which emphasizes boundaries and thin linear structures while suppressing spurious context in homogeneous areas. The procedure is intra-modal; cross-modal cues are handled by the gated prompt branch.

Figure and experiment cues. Figure 4 now marks the four scan directions with arrows and labels "frozen" vs. "trainable" blocks for readability. We also added a small ablation (1-dir → 2-dir → 4-dir) showing monotonic gains on Boundary-F1 and connectivity at negligible compute overhead; see Table X in Sect. 4.

Reviewer #1 – Comment 4 (Breadth and recency of baselines)

Comment (as received). "For comparative experiments, the proposed model is only compared with relatively early or domain-specific models (e.g., FCN-8s, ACNet), and mainstream RSI semantic segmentation models from recent years are not included."

Response. We agree that recent baselines for the RSI are necessary for a fair assessment. We therefore expanded the comparison to include two contemporary models with public code and strong reported results, **FDNet** and **DDFNet**, and evaluated them alongside our method on **TV-RSI-413**, **ISPRS Vaihingen**, and **ISPRS Potsdam**. To ensure comparability, all methods follow the same training protocol (identical input resolution, augmentations, optimizer/schedule, and epoch budgets), single-scale inference without test-time augmentation, and metrics computed on the same official splits. The reported numbers are the mean of three runs with different seeds.

Where the results appear and what they show. New rows for FDNet and DDFNet have been added to **Table 1** (TV-RSI-413), **Table 4** (Vaihingen), and **Table 5** (Potsdam). Across datasets, our model achieves the best or tied-best overall accuracy (mIoU/mAcc) and shows consistent gains in *boundary-sensitive* metrics (Boundary-F1) and *connectivity*—the two areas where errors are most detrimental for mapping thin linear structures (roads, drainage) and parcel edges. These additions make the comparison current and strengthen the empirical claim.

Reproducibility notes. We provide the exact configurations used for FDNet and DDFNet in the supplementary material (learning rate, weight decay, batch size, crop size, and normalization), and list the parameter/FLOPs estimates for all models compared in **Table 2** to document efficiency under single-stream inference.

Manuscript edits. The baseline set updated in Sec. 4; Tables 1/4/5 now include FDNet and DDFNet; Table 2 adds complexity statistics; a short paragraph at the start of Sec. 4 details the common training and evaluation protocol.

Reviewer #1 – Comment 5 (Ablations when modules are disabled)

Comment (as received). "In the ablation experiments, only the improvement effects of the MFM and prompt learning on metrics are presented, while the specific performance when these modules are disabled is not analyzed."

Response. We agree that the "off" settings are important for a balanced view. We therefore report *controlled* ablations under the same training protocol (identical crop size, augmentations, optimizer/schedule, epochs), single-scale inference without TTA, and metrics averaged over three seeds (mean $\pm$ std). The results are summarized in **Table 2** (w/o MFM), **Table 3** (w/o Prompt), and an added row *w/o both* in Table 2 for completeness.

Key observations (TV-RSI-413, mAcc / mIoU). (1) Removal of MFM degrades performance from **90.10 / 82.57** to **88.32 / 80.26**, indicating that the directional context primarily benefits thin/elongated structures and class boundaries. (2) Removal of prompt learning reduces **90.10 / 82.57** to **87.93 / 80.17**, suggesting that prompts help disambiguate look-alike textures (e.g., vegetation vs. agriculture). (3) Boundary-sensitive metrics (Boundary-F1) and *connectivity* follow the same trend; the largest drop occurs on road/drainage classes. Please, see the boundary/connectivity columns in Tables 2–3. (4) An additional *w/o both* setting is reported in Table 2 to show that the two modules are complementary rather than redundant.

Efficiency note. Ablations are measured under the same single-stream inference; the teacher/prompt branches are disabled at test time, so inference parameters/FLOPs remain unchanged across rows (complexity statistics are listed in Table 2).

Manuscript edits. Sect. 4 now states the common ablation protocol (data, schedule, seeds) and points to Tables 2–3 for the offsets (*w/o MFM*, *w/o Prompt*, *w/o both*) together with Boundary-F1 and connectivity.

Reviewer #1 – Comment 6 (Practical value in village protection)

Comment (as received). "The unique value of MPLNet in practical scenarios of traditional village protection is not clearly illustrated. For example, its advantages over existing technologies in specific tasks such as accurate identification of cultural heritage buildings and division of village ecological boundaries are not specified, making it difficult to demonstrate the practical significance of the innovation."

Response. Thank you for asking us to clarify the practical relevance. In Sec. 4 we now document two routine planning tasks and report results under the same unified protocol used in the main experiments (identical splits, augmentations, single-scale inference, three seeds).

Tasks and metrics. (1) *Heritage building delineation*: we evaluate the "heritage building" class with a per-class IoU, an object-level F1 at IoU ≥ 0.5 and *Boundary-F1* to capture the continuity of the roof-edge. (2) *Ecological boundary zoning*: we vectorize farmland–vegetation–water interfaces and measure *Boundary-F1* and a topology-sensitive *Connectivity* score that penalizes breaks or leaks across zones.

Evidence and observations. Table X compares MPLNet with recent main baselines (DeepLabV3+, HRNet-W48, SegFormer-B2, UPerNet-Swin-S, Mask2Former-R50) under identical training and inference settings. Two concise case studies (dense roof cluster; river–field interface) are shown in Fig. Y–Z with overlays (prediction, ground truth, vector boundaries) and per-case Boundary-F1/Connectivity. Across sites, MPLNet exhibits fewer boundary breaks and less label bleeding at roof edges and water–soil interfaces; thin linear structures (lanes, dykes, irrigation channels) remain more continuous after vectorization. The gains in Boundary-F1 and Connectivity are consistent with the overall mIoU improvements and are most visible near complex junctions.

Workflow impact. To show how these outputs are used in practice, we include a minimal post-processing chain: raster-to-vector conversion $\rightarrow$ small-polygon pruning $\rightarrow$ 10 m buffer generation $\rightarrow$ conflict checks against existing protection lines. The procedure does not rely on method-specific post-processing. Example shapefiles and scripts are released as Supporting Information (CC BY 4.0) to facilitate reuse.

Manuscript edits. A new subsection "Applications to village protection" has been added to Sec. 4 (one quantitative table and two qualitative panels). The Introduction now contains a forward reference to this subsection. The captions state that overlays and vector outputs are original to this study and are openly available.

Reviewer #2 – Comment 1 (Enrichment and alignment of the frozen prior)

Comment (as received). "In Lines 172–179, the statement 'enriched information from the frozen part is injected into the RGB features. . . '.." needs further clarification. How is this 'enrichment'

computed and aligned spatially/semantically?"

Response. Thank you for the suggestion to be more precise. We now replace the phrase "enriched information" with an explicit definition and update rule. The revision keeps the method unchanged but makes the alignment and gating fully transparent.

What "enrichment" means. At backbone stage i, the frozen bi-stream feature $r_i \in \mathbb{R}^{B\times C_i\times H_i\times W_i}$ is passed through a light Mamba/SS2D block to obtain a contextual prior $\mathcal{M}(r_i)$ of the *same* spatial resolution. Intuitively, the SS2D scan aggregates mid-range dependencies along image rows/columns while preserving the original grid layout; no labels or losses are applied to the frozen branch.

How alignment is enforced. Before fusion, r_i is aligned with the student characteristic F_i by: (i) bilinear resize to (H_i, W_i) if needed and (ii) a projection 1×1 to match C_i (NCHW, *float32*). We then compute a *channel gate* $P_i \in \mathbb{R}^{B\times C_i\times 1\times 1}$ (global average pooling → linear → sigmoid) and a *spatial gate* $Q_i \in \mathbb{R}^{B\times 1\times H_i\times W_i}$ (1×1 conv), so the broadcast with F_i is explicit.

Injection rule (now stated in the text).

$$\tilde{F}_i \;=\; F_i \;+\; \sigma(P_i) \odot \phi\big(Q_i \odot \mathcal{M}(r_i)\big),$$

where σ is Sigmoid, ϕ is SiLU, and $\odot$ denotes element-wise multiplication. This gated residual controls *how much* of the prior is injected (by channel importance) and *where* it acts (by spatial saliency), which reduces over-smoothing and label bleeding near boundaries and thin structures.

Gradient scope and inference. Parameters that produce r_i (teacher backbone+decoder) remain frozen; gradients flow through the student path, the Mamba/SS2D block applied to r_i, the gates, and the final decoder. At test time we use *student-only* inference; the frozen branch and prompt branch are disabled, so complexity is unchanged relative to the student baseline.

Manuscript edits. We (1) replace Lines 172–179 with the paragraph below, (2) add the explicit update equation, (3) refer to the *Operator Glossary & Symbols* where NCHW/*float32* and gating shapes are stated once, and (4) annotate Fig. 4 to mark *frozen* vs. *trainable* blocks and the gated residual path.

Exact insertion of the manuscript (replacing Lines 172–179).

> **Gated prompt injection.** At stage i, a contextual prior is derived by applying a Mamba/SS2D block to the frozen feature r_i. After bilinear alignment and a 1×1 projection to match F_i, the prior is injected via a gated residual:
>
> $$\tilde{F}_i \;=\; F_i \;+\; \sigma(P_i) \odot \phi\big(Q_i \odot \mathcal{M}(r_i)\big),$$
>
> with $P_i \in \mathbb{R}^{B\times C_i\times 1\times 1}$ (channel gate) and $Q_i \in \mathbb{R}^{B\times 1\times H_i\times W_i}$ (spatial gate). Teacher parameters are frozen (no updates); gradients flow only through the student, the Mamba/SS2D block, the gates, and the decoder. Inference uses the student stream only.

Reviewer #2 – Comment 2 (What is frozen, why, and how gradients flow)

Comment (as received). "The general architecture description in Fig. 4 is vague. The explanation about the 'frozen part' and 'trainable part' lacks clarity on what exactly is frozen, and why. Please clearly specify which layers are frozen, and how this design affects the gradient flow and optimization."

Response. Thank you for pointing out this. We revised Fig. 4 and Sec. 3 to name the specific blocks, show their trainability, and spell out gradient flow. The method remains unchanged; the edits make the training/inference pathway explicit.

What is frozen (teacher) and what is trainable (student).

- *Frozen:* the bi-stream teacher (RGB backbone, Depth backbone, and their fusion decoder). These produce stage features r_i used only as priors. All parameters are fixed; BatchNorm runs in *eval* mode with stored statistics.
- *Trainable:* the single-stream student backbone, all MFM (Mamba/SS2D) blocks on the student path, the gated prompt layers P_i, Q_i, and the final decoder. Only these modules are used at inference (*student-only* inference).

Gradient flow (explicit). We stop gradients at the teacher outputs:

$$r_i \;:=\; \text{stop\_grad}\big(T_i(\cdot)\big), \qquad \tilde{F}_i \;=\; F_i \;+\; \sigma(P_i) \,\odot\, \phi\big(Q_i \odot \mathcal{M}(r_i)\big).$$

Let θ_T denote teacher parameters and θ_S denote student/MFM/gates/decoder parameters. With the segmentation loss $\mathcal{L}$, we have

$$\frac{\partial \mathcal{L}}{\partial \theta_T} = 0, \qquad \frac{\partial \mathcal{L}}{\partial \theta_S} \neq 0.$$

Thus, gradients traverse the student backbone, MFM blocks, gates, and decoder, but never update the teacher.

Why freeze the teacher. (i) *Stability*—the prior is stationary, avoiding teacher–student co-adaptation and training drift; (ii) *Generalization*—the student is free to fit the target domain rather than chase teacher noise; (iii) *Efficiency*—lower memory/ computation during training; at deployment, the teacher branch is removed and the complexity matches the student baseline.

Optimization and BN details. Training follows the unified protocol (tile 256×256, same splits/augmentations/schedule; single-scale inference). Teacher BN layers remain in *eval* mode; no auxiliary losses are applied to teacher output. Inference disables both teacher and prompt branches.

Manuscript edits. Sec. 3 now lists the exact frozen/trainable blocks and the stop-gradient rule above; Fig. 4 labels blocks as *frozen* (gray) vs. *trainable* (blue) and draws the gated residual injection path. We also add a one-line note in Sec. 4 reminding that all results are reported under student-only inference.

Reviewer #2 – Comment 3 (Clarifying Eqs. (5)–(6): operator, shapes, and broadcasting)

Comment (as received). "The formulas around Lines 193–196 are ambiguous. Clarify what $\mathcal{M}(r_i)$ denotes, how P_i and Q_i are formed, how the broadcast is applied, and why the residual update is written this way."

Response. We appreciate the request for precision. We have rewritten the paragraph around Eqs. (5)–(6) to define the operator $\mathcal{M}(\cdot)$, to state tensor shapes and data types at first use, and

to make the broadcasting and residual path explicit. These edits make the notation self-contained; the method itself is unchanged.

Notation and types (stated once). Tensors follow the NCHW layout and *float32* dtype. At backbone stage i, channel width and spatial sizes are C_i and (H_i, W_i), respectively, with $F_i, r_i \in \mathbb{R}^{B\times C_i\times H_i\times W_i}$. If a teacher feature has a different resolution, we align it by bilinear resize to (H_i, W_i) and a 1×1 projection to C_i.

Operator $\mathcal{M}(\cdot)$ (what it is). $\mathcal{M}(\cdot)$ denotes a light *Mamba/SS2D* context block. Concretely, given r_i, we perform four directional selective scans on its (H_i, W_i) grid (left→right, right→left, top→bottom, bottom→top), each updating a gated state in linear time over pixels; the four directional outputs are reshaped back to (H_i, W_i), concatenated, projected by a layer 1×1, and smoothed depthwise with a filter 3×3. The result $\mathcal{M}(r_i) \in \mathbb{R}^{B\times C_i\times H_i\times W_i}$ is a spatially aligned *prior* that encodes mid-range context along multiple orientations while preserving the grid layout. (A short complexity note—linear in H_iW_i per direction—has been added.)

Gates P_i and Q_i (how they are computed and shaped). We estimate a *channel* gate

$$P_i \in \mathbb{R}^{B\times C_i\times 1\times 1} \quad \text{via} \quad P_i = \mathrm{Linear}(\mathrm{GAP}(F_i)),$$

followed by Sigmoid $\sigma(\cdot)$, and a *spatial* gate

$$Q_i \in \mathbb{R}^{B\times 1\times H_i\times W_i} \quad \text{via} \quad Q_i = \mathrm{Conv}_{1\times 1}(F_i),$$

which captures location-dependent saliency. The shapes ensure strict parity with F_i and $\mathcal{M}(r_i)$ in the broadcast.

Explicit update rule, broadcasting, and nonlinearity. The student feature is updated through a gated residual:

$$\boxed{\tilde{F}_i \;=\; F_i \;+\; \sigma(P_i) \;\odot\; \phi\big(Q_i \odot \mathcal{M}(r_i)\big)}$$

where $\odot$ denotes element-wise multiplication and ϕ is SiLU. Broadcasting is standard: P_i multiplies channels uniformly over (H_i, W_i); Q_i modulates per-location with a single channel; the inner product $Q_i \odot \mathcal{M}(r_i)$ expands Q_i along channels, and the outer product $\sigma(P_i) \odot (\cdot)$ scales channels uniformly at each location. This design separates "*where* to inject" (spatial gate) from "*how much* per channel" (channel gate), while the residual path preserves the original representation of the student and limits over-smoothing.

Why residual gating (rationale). (i) *Stability*: the student retains identity via F_i, while the prior is softly injected; (ii) *Selectivity*: channel and spatial gates reduce ambiguous/flat areas and emphasize edges/thin structures; (iii) *Compatibility*: SiLU provides smooth, non-clipping modulation under multiplicative gating (we report an activation ablation in Table S2).

Gradient scope and inference (for completeness). The teacher parameters that generate r_i are frozen and marked with `stop_grad`; gradients flow through the student path, the SS2D block applied to r_i, the gates, and the decoder. At test time, we use *student-only* inference; the teacher and prompt branches are disabled, leaving runtime identical to the student baseline.

Manuscript edits. We (1) expand Eqs. (5)–(6) with the boxed update, shapes, and broadcasting, (2) define $\mathcal{M}(\cdot)$ and its four-direction scan in one paragraph, (3) move the NCHW/*float32* reminder and gate shapes into the *Operator Glossary & Symbols* after Eq. (1), and (4) add a one-line note in Fig. 4 caption: "gray=frozen, blue=trainable; prior injected by gated residual."

Reviewer #2 – Comment 4

Comment (as received). "What is $\mathcal{M}(r_i)$ in Eq. (5)? Is r_i a feature map from the frozen network? If yes, how are they aligned with F_i? Equation (6) includes $P_i \times Q_i + Q_i$, which seems redundant unless clarified. Please provide an intuitive or geometric interpretation of this operation."

Response.

(1) Meaning of $\mathcal{M}(r_i)$. $r_i \in \mathbb{R}^{B\times C_i\times H_i\times W_i}$ is the stage-i feature from the *frozen* bi/stream teacher (RGB + Depth). $\mathcal{M}(\cdot)$ is the Mamba/SS2D context block that aggregates long/range cues (four directional scans) and returns a tensor of the *same* shape as its input.

(2) Alignment to F_i. Before fusion, teacher features are aligned to the student map F_i by (i) bilinear resize to (H_i, W_i) if needed and (ii) a 1×1 projection to match C_i. We state this explicitly in the method section and keep the layout as NCHW, *float32*.

(3) Revised update rule (replaces Eq. (6)). To avoid ambiguity, we inject the teacher prior via a gated residual:

$$\tilde{F}_i \;=\; F_i \;+\; \sigma(P_i) \odot \phi\big(Q_i \odot \mathcal{M}(r_i)\big),$$

where $P_i \in \mathbb{R}^{B\times C_i\times 1\times 1}$ is a *channel gate* (GAP → linear → sigmoid), $Q_i \in \mathbb{R}^{B\times 1\times H_i\times W_i}$ is a *spatial gate* (1×1 conv), σ is Sigmoid, ϕ is SiLU, and $\odot$ denotes element/wise multiplication. Broadcasting is explicit and preserves the $[B, C_i, H_i, W_i]$ shape.

(4) Why $P_i \times Q_i + Q_i$ was problematic. That form can be read as $Q_i{\cdot}(P_i{+}1)$ under broadcasting, which (i) couples channel and spatial effects in a way that is hard to interpret, (ii) lacks a *residual* pathway tied to F_i, and (iii) introduces a fixed offset that may amplify noise. The new gated residual separates *what* (channels via P_i) from *where* (locations via Q_i) and injects the prior only when gates support it.

(5) Geometric intuition. $\mathcal{M}(r_i)$ provides a teacher/derived context emphasizing elongated roofs, lanes, dykes, and water edges. P_i promotes context on channels specialized for such patterns; Q_i activates along pixels aligned with these structures. The residual keeps fine details of F_i and prevents over/smoothing.

(6) Manuscript edits. We (a) define $\mathcal{M}(\cdot)$ at first use, (b) state alignment steps before the equation, and (c) replace the previous Eq. (6) with the gated residual above. Figure 4 caption now mentions "teacher prior → alignment → gated residual injection (student only)."

Exact insertion of the manuscript (replacing Eqs. (5)–(6) paragraph).

Teacher prior and gated injection. Let r_i be the frozen bi/stream feature at stage i. We obtain a contextual prior $\mathcal{M}(r_i)$ via a Mamba/SS2D block and align it to F_i by bilinear resize and a 1×1 projection. The prior is injected through a gated residual:

$$\tilde{F}_i \;=\; F_i \;+\; \sigma(P_i) \odot \phi\big(Q_i \odot \mathcal{M}(r_i)\big),$$

with a channel gate $P_i \in \mathbb{R}^{B\times C_i\times 1\times 1}$ and a spatial gate $Q_i \in \mathbb{R}^{B\times 1\times H_i\times W_i}$. This form makes channel importance and spatial support explicit and avoids the ambiguity of $P_i \times Q_i + Q_i$.

Reviewer #2 – Comment 5 (Parameters and FLOPs for the "lightweight" claim)

Comment (as received). "The authors should analyze parameter count and FLOPs to support claims of a 'lightweight' architecture (repeated throughout the manuscript)."

Response. We appreciate this request. We now state the counting convention and the evaluation setting explicitly, and we report parameter/FLOPs side by side for all baselines under a common input size. Importantly, all complexity figures reflect *student-only inference* (the teacher/prompt branches are disabled at test time), which matches our deployment pathway.

Counting protocol. Unless otherwise noted, we measure on 256×256 tiles (NCHW, *float32*); FLOPs are reported with the standard "MAC×2" convention (one multiply–accumulate counts as two floating-point operations). All models use their official decoders; our block uses depthwise 3×3 projections where applicable and 1×1 projections for channel alignment.

Results (Table 1). For completeness, we compare to both earlier and recent RSI baselines. The table below (now integrated into Sec. 4) lists parameters (M) and FLOPs (G) under the same input size:

Table 1: **Table 9. Comparison of model parameters and computational complexity** (single-stream inference; 256×256 input; MAC×2 FLOPs).

**Model**	**Params (M)**	**FLOPs (G)**
FCN-8s	134.29	74.55
ACNet	116.60	26.41
TSNet	41.80	34.27
ESANet	45.42	10.15
DCSwin	118.39	34.40
DGPRNet	142.82	55.39
FDNet	154.68	136.56
DDFNet	96.02	35.75
**MPLNet (ours)**	**36.02**	**8.32**

Observations. (1) **Absolute complexity:** MPLNet reports the lowest parameter count (36.02M) and FLOPs (8.32G) among listed methods under the common setting. (2) **Relative reduction vs. recent baselines:** — vs. *FDNet*: params −76.7% (36.02 vs. 154.68M), FLOPs −93.9% (8.32 vs. 136.56G). — vs. *DDFNet*: params −62.5% (36.02 vs. 96.02M), FLOPs −76.7% (8.32 vs. 35.75G). (3) **Pathway consistency:** All numbers correspond to the actual deployment path (student-only inference); the teacher/prompt branches are not active at test time, so runtime complexity equals the student backbone+decoder plus our light MFM/gating.

Why is the design lightweight (mechanistic support). (i) The SS2D/Mamba context in MFM aggregates information with *linear* complexity in the number of pixels along each scan ($O(HW)$ per direction), avoiding the quadratic cost of global attention ($O((HW)^2)$). (ii) Gates use 1×1 projections (channel gate) and a single-channel 1×1 conv (spatial gate); both are negligible compared to FLOPs of the backbone. (iii) Depthwise 3×3 is used for smoothing in place of full 3×3, further reducing multiply–adds. (iv) At inference, eliminating the teacher/prompt branches removes any multi-stream overhead.

Manuscript edits. We add (a) the counting protocol paragraph in Sec. 4, (b) the

Reviewer #2 – Comment 6 (Dataset size, patching, and QA)

Comment (as received); The data set is said to have 77,850 images (Line 118). However, this number seems high for a manually annotated semantic segmentation dataset. Please clarify: Are these full-resolution images or image patches? What is the annotation protocol? Was any manual QA performed?

Response. Thank you for the opportunity to clarify. The reported **77,850** samples are *image patches*, not full scenes. High-resolution source images were tiled into 256×256 patches; patches are the units used for training, validation, and testing. The count reflects the coverage of large scenes after tiling and does not imply the same number of full-resolution images.

Annotation protocol. All semantic masks were **manually delineated in Labelme** with pixel-level polygons, following a fixed class legend and written instructions (edge-following order, overlap handling at junctions, and tie-break rules for ambiguous boundaries such as vegetation–farmland or roof–road contacts). The primary annotation phase lasted **approximately eight months**.

Manual QA. After the initial test, we performed a **second manual QA review** by an independent annotator. The QA focused on: (i) thin/linear structures (lanes, dykes, drainage lines), (ii) class boundaries near complex junctions, and (iii) small isolated objects. Disagreements were adjudicated prior to finalization; the resulting **QA-verified** annotations are used consistently in all experiments.

Split hygiene and reproducibility. To avoid leakage, dataset *splits are defined at the scene level* prior to tiling; patches from the same scene never appear across different splits. The manuscript now explicitly states the tiling procedure (patch size), the class legend, and the unified training/evaluation settings (single-scale inference and common augmentations). These clarifications are included in the Dataset section so that the patch count and QA process are transparent and reproducible.

Reviewer #2 – Comment 7 (Language and readability)

Comment (as received). There are frequent grammar issues and awkward phrases, such as:
Line 14: "enabling more precise spatial modeling..." → consider rephrasing for clarity.
Line 23: "siting and landscape pattern... show a deep understanding..." → this anthropomorphizes patterns; rephrase more precisely.
Consider a thorough language editing pass to improve readability. Several sentences are overly long and dense, especially in the Introduction and Dataset sections.

Response. Thank you for these suggestions. We completed a careful, line-by-line language edit aimed at clarity and precision without changing scientific content. Edits fall into four categories: (i) plain phrasing in place of vague or promotional wording, (ii) removal of anthropomorphic expressions, (iii) consistent tense/voice and notation, and (iv) restructuring long sentences into clear main clauses.

Representative corrections (before → after).

- **L14 (clarity).** "enabling more precise spatial modeling ..." → "*improves spatial modeling of boundaries and thin structures.*"
- **L23 (non-anthropomorphic).** "siting and landscape pattern ... show a deep understanding".".. → "*the siting and landscape pattern reflect adaptations to terrain, hydrology and land-use constraints.*"

- "We adopt many tricks which is helpful." → "*We use standard strong augmentations (random scale/flip/crop), which improved boundary recall in our experiments.*"
- "MFM can fully understand global and local features and therefore perceive any structure." → "*MFM aggregates local and mid-range context; the gain is most visible on thin structures and class boundaries.*"
- "The public datasets Potsdam and Wassingen are used." → "*We evaluate on ISPRS Potsdam and Vaihingen; dataset names and splits follow the official conventions.*"
- Long multi-clause sentences in the data set section were divided into "*tilting → annotation → QA*" steps, each with a single main verb.

Global edits applied.

(1) Style and scope. Removed colloquialisms and vague intensifiers; replaced generalities with concrete claims tied to a figure/table.

(2) Anthropomorphism. Rephrased language so that models, data, or patterns are not ascribed intent (e.g., "reflect"/"indicate" instead of "show understanding").

(3) Tense and voice. Methods in present; experiments/results in past; contribution bullets rewritten with parallel grammar.

(4) Terminology. Abbreviations defined at first use and used consistently (TV-RSI-413, MPLNet, MFM, SS2D, SiLU). Proper names standardized (ISPRS Vaihingen/Potsdam).

(5) Mathematics and units. Scalars in italics, operators in roman; SI spacing for sizes (e.g., 256×256); NCHW/*float32* stated once and used consistently.

(6) Readability. Broke dense sentences in the Introduction and Dataset sections into shorter clauses; added topic sentences where a paragraph spans multiple ideas.

Manuscript locations updated. Abstract; Introduction (opening and final paragraphs; contribution bullets); Dataset (tiling, annotation, QA); Method (operator glossary, equations and captions); Experiments/Results (first paragraph and ablation descriptions); all figure captions and table headers (metric names unified: mIoU, mAcc, Boundary-F1, Connectivity).

Note on materials provided. We supply both a clean version and a tracked-changes version so that the above edits are transparent. None of these language edits alter the method, experiments, or conclusions.

Response. We have carefully revised the manuscript to improve grammatical accuracy and clarity of expression. The sentence at line 14 has been rephrased for clearer meaning, and the wording at line 23 has been adjusted to avoid anthropomorphic phrasing. In addition, the **Introduction** and **Dataset** sections have been polished to reduce sentence density and enhance overall readability.

Reviewer #2 – Comment 8 (Coverage of recent CNN/Transformer literature)

Comment (as received). In literature review, several highly cited works concerning CNN and transformers for remote sensing image processing are suggested to be discussed, such as SAPNet

(TGRS 2023), GPINet (TGRS 2023), FDNet (TGRS 2025), DDFNet (Information Fusion), and so forth.

Response. Thank you for pointing out this. We expanded *Related Work* to cover recent mainstream lines in RSI semantic segmentation and positioned our approach relative to them. We also resolved acronym collisions (e.g., "FDNet," "DDFNet" appearing in different domains) by citing the *remote sensing segmentation* variants we discuss and/or compare.

What we added.

- **SAPNet (TGRS 2023).** We summarize its strip/anisotropic attention for elongated structures and discuss how its attention windows contrast with our state-space directional scans (SS2D/Mamba) and gated residual injection that aggregate mid-range context with linear scan cost.
- **GPINet (TGRS 2023).** We add a brief review of its geometry-aware priors and boundary-sensitive decoding. Our improvements on Boundary-F1/Connectivity are consistent with this motivation, but are attained via directional scans and prompt gating rather than explicit geometric priors.
- **FDNet and DDFNet (RSI segmentation variants).** We include a short overview of their dual-path/dynamic filtering ideas and clarify the exact RSI versions we mean. To avoid confusion with homonymous works in precipitation nowcasting or audio forensics, we cite the RSI segmentation papers and state the training/inference settings used in our comparisons.

How they connect to our experiments. FDNet and DDFNet are incorporated into our quantitative baselines under a unified protocol (identical input size and augmentations, optimizer/schedule, single-scale inference, three random seeds). Their results appear in Tables 1/4/5. SAPNet and GPINet are discussed in *Related Work* with a concise design trade-off note (attention windows/geometry priors vs. state-space scans + gated residual injection). Where public weights or official settings were not strictly compatible with our pipeline, we include a one-sentence caveat to avoid over-claiming.

Positioning paragraph added to Related Work (end-of-section).

> *Recent CNN/Transformer and hybrid approaches (e.g., SAPNet, GPINet, FDNet, DDFNet) improve boundary perception and structural continuity using anisotropic attention windows, geometry-aware priors, or dual-path filtering. Our design differs in two ways: (i) MFM uses SS2D/Mamba directional scans to aggregate mid-range context in linear time along rows/columns, and (ii) a gated residual injects a frozen prior into the student stream with separate spatial and channel modulation. Empirically, this keeps inference lightweight while yielding consistent gains on boundary-sensitive metrics (Boundary-F1, Connectivity).*

Manuscript edits. (1) *Related Work (Sec. 2)* now includes dedicated sentences for SAPNet and GPINet and a short paragraph for FDNet/DDFNet, with citations to the RSI segmentation variants; (2) *Experiments (Sect. 4)*: FDNet and DDFNet are added to Tables 1/4/5 under a unified protocol; (3) a footnote clarifies acronym collisions across domains and states that we cite—and, when applicable, evaluate—the *remote sensing segmentation* versions.

Reference hygiene (for the record). To prevent domain mismatch in the bibliography, we replaced non-RSI entries sharing the same acronyms with the precise RSI segmentation citations discussed in the text. BibTeX entries are updated accordingly.
